# 1-Deoxysphingolipids, Early Predictors of Type 2 Diabetes, Compromise the Functionality of Skeletal Myoblasts

**DOI:** 10.3389/fendo.2021.772925

**Published:** 2021-12-24

**Authors:** Duyen Tran, Stephen Myers, Courtney McGowan, Darren Henstridge, Rajaraman Eri, Sabrina Sonda, Vanni Caruso

**Affiliations:** ^1^ School of Pharmacy and Pharmacology, College of Health and Medicine, University of Tasmania, Hobart, TAS, Australia; ^2^ School of Health Science, College of Health and Medicine, University of Tasmania, Launceston, TAS, Australia; ^3^ Sport Performance Optimization Research Team, School of Health Sciences, College of Health and Medicine, University of Tasmania, Launceston, TAS, Australia; ^4^ Institute for Research on Pain, Istituto di Formazione e Ricerca in Scienze Algologiche (ISAL) Foundation, Rimini, Italy

**Keywords:** 1-deoxysphingolipids, glucose uptake, myoblasts, myoblast differentiation, autophagy, myotubes, type 2 diabetes mellitus

## Abstract

Metabolic dysfunction, dysregulated differentiation, and atrophy of skeletal muscle occur as part of a cluster of abnormalities associated with the development of Type 2 diabetes mellitus (T2DM). Recent interest has turned to the attention of the role of 1-deoxysphingolipids (1-DSL), atypical class of sphingolipids which are found significantly elevated in patients diagnosed with T2DM but also in the asymptomatic population who later develop T2DM. *In vitro* studies demonstrated that 1-DSL have cytotoxic properties and compromise the secretion of insulin from pancreatic beta cells. However, the role of 1-DSL on the functionality of skeletal muscle cells in the pathophysiology of T2DM still remains unclear. This study aimed to investigate whether 1-DSL are cytotoxic and disrupt the cellular processes of skeletal muscle precursors (myoblasts) and differentiated cells (myotubes) by performing a battery of *in vitro* assays including cell viability adenosine triphosphate assay, migration assay, myoblast fusion assay, glucose uptake assay, and immunocytochemistry. Our results demonstrated that 1-DSL significantly reduced the viability of myoblasts in a concentration and time-dependent manner, and induced apoptosis as well as cellular necrosis. Importantly, myoblasts were more sensitive to the cytotoxic effects induced by 1-DSL rather than by saturated fatty acids, such as palmitate, which are critical mediators of skeletal muscle dysfunction in T2DM. Additionally, 1-DSL significantly reduced the migration ability of myoblasts and the differentiation process of myoblasts into myotubes. 1-DSL also triggered autophagy in myoblasts and significantly reduced insulin-stimulated glucose uptake in myotubes. These findings demonstrate that 1-DSL directly compromise the functionality of skeletal muscle cells and suggest that increased levels of 1-DSL observed during the development of T2DM are likely to contribute to the pathophysiology of muscle dysfunction detected in this disease.

## Introduction

1-deoxysphingolipids (1-DSL) are a class of atypical sphingolipids found significantly elevated in plasma of individuals with impaired fasting glucose, metabolic syndrome (MetS) and T2DM (1, 2). Interestingly, recent clinical studies demonstrated that plasma 1-DSL are also significantly elevated in non-diabetic individuals who later develop T2DM (3). In contrast, these atypical lipids are not elevated in patients with diabetes type 1 (T1DM) (4). This suggests that 1-DSL could be considered early predictors of T2DM independently of the levels of glycated haemoglobin and MetS in general population (3, 5). Recent studies revealed that these atypical lipids were found significantly elevated in obese and T2DM patients compared to athletes and lean individuals, and that their accumulation appeared to cause insulin resistance *in vitro* (5). However, the underlying molecular mechanisms leading to development of T2DM in patients with elevated atypical sphingolipids have not been fully elucidated.

The first step in *de novo* sphingolipid synthesis occurs in the endoplasmic reticulum (ER) where the enzyme serine palmitoyltransferase (SPT) catalyses the condensation of serine and palmitoyl Co-A to form sphinganine (SA) which is then metabolised to ceramide and complex sphingolipids (sphingomyelins, glycosphingolipids) (6). On the other hand, when alanine is used over serine, SPT sintethises the atypical sphingolipids 1-DSL. This class of sphingolipids lacks the C1-OH group of canonical sphingolipids which make them resistant to normal sphingolipid catabolism thus resulting in the accumulation of 1-DSL inside cells and tissues (7). Recent *in vitro* studies have demonstrated accumulation of 1-DSL promote death of pancreatic beta-cells and interfere with insulin secretion (8) and may be involved in the mechanisms of insulin resistance (5). Another clinical study showed that 1-DSL levels are elevated in the early stages of diabetic neuropathy, although this elevation did not correlate with the clinical course of the disease (9).

Deterioration of skeletal muscle mass is a hallmark of patients with T2DM (10). A reduction in skeletal muscle’s ability to respond to insulin and the subsequent development of insulin resistance are key events in the establishment of high blood glucose levels and impaired glycaemic control. In addition to these metabolic dysfunctions, muscle loss or atrophy is associated with diminished strength, quality of life, and early mortality (11, 12). T2DM-generated muscle atrophy is initiated by dysfunctions in the activity of myogenic progenitor cells, so-called myoblasts (13–15). Specifically, proliferation, migration, and differentiation of myoblasts, which are vital for the maintenance of skeletal muscle integrity, are reduced during T2DM (16, 17). While muscle dysfunction and atrophy coincide with the development of T2DM, the exact relationship between T2DM and the reduced functionality of myoblasts remains largely unexplored.

Given the cytotoxic properties associated with atypical lipids, the aim of this study was to investigate whether 1-DSL compromises the functionality of skeletal myoblasts and their differentiation into mature myotubes thus contributing to the pathophysiology of T2DM.

## Materials and Methods

### Mammalian Cell Culture

Immortalised mouse C2C12 myoblast cell line (ATCC, CRL-1772, USA) was cultured in a complete medium comprising high glucose Dulbecco’s Modified Eagle’s Medium (DMEM) – (Sigma, D6429, USA), 10% Fetal Bovine Serum (FBS) (Thermo Fisher Scientific, 10099158, USA), and 1X penicillin-streptomycin (Thermo Fisher Scientific, 10378016, USA) at 37°C, 5% CO2, and saturating humidity. The cells were passaged once a week before reaching confluence using Trypsin – EDTA solution (Sigma, T4049, USA).

To induce myoblast differentiation into myotubes, the complete medium was changed to a differentiation medium of DMEM with 2% horse serum (Thermo Fisher Scientific, 260500, USA) and 1X penicillin-streptomycin for 5 days. Lipid treatments included either 1-deoxysphinganine (Avanti Polar Lipids, 860493P, USA) or the control non-toxic - sphinganine (Avanti Polar Lipids, 860498P, USA), or the control toxic - palmitic acid (Sigma, P0500, USA) which causes insulin resistance in skeletal muscle cells (18). Lipids were dissolved in absolute ethanol to obtain a stock concentration of 1 mM. Fatty acid-free - bovine serum albumin (Sigma, A6003, USA) was used as a lipid carrier and added to the complete medium at the same molar concentration as the lipids. The working concentrations for lipids were prepared by diluting lipid stock solutions in a complete medium or differentiation medium according to the relevant assays.

### Cell Viability Assay

C2C12 cells were seeded into 96-well plates (2x10^4^ cells/well) and lipids at concentrations of 0.5 µM, 1 µM, and 3 µM, were added when the cells reached 100% confluency, as described in the figure legends. The numbers of live and dead cells were quantified by staining with Trypan Blue dye (Life Technologies, 5250061, USA) and manual counting was performed using a Neubauer chamber (Blau Brand, 717810, Germany). Alive cells as indicated by no Trypan blue staining were counted under a microscope and our read-out normalised to this number.

Cell viability was also quantified by Thiazolyl Blue Tetrazolium Bromide (MTT) assay. C2C12 cells (2x10^4^ cells/well) were cultivated in 96 well plates and treated with the lipids as described in the figure legends. After the treatment period, 10 μL of 5 mg/mL MTT (Sigma, M5655, USA) was dissolved in PBS and was added to each well, and incubated for 2 hours at 37°C. After aspirating the supernatant, formazan crystals were dissolved in 100 µl of DMSO and quantified by measuring the absorbance at 570 nm and reference at 670 nm using the Infinite 200 Pro microplate reader (Tecan, 396235, Switzerland).

### ATP Assay

Intracellular Adenosine Triphosphate (ATP) levels at different time points were quantified using the CellTiter-Glo Luminescent Viability Assay (Promega, G7570, USA), following the manufacturers’ instructions. Luminescence was recorded over an integration period of 0.25 to 1 second using the Infinite 200 Pro microplate reader. ATP levels for each cell were calculated by normalizing the luminescence levels to the number of live cells After lipid treatment, wells were washed with 1X PBS, then 50 μL of Trypsin was added to the wells and incubated for 10 min. Trypsin reaction was stopped by adding 60 μL fresh 10% FBS DMEM (total volume = 110 μL). 10 μL of cell suspension was mixed with 10 μL of Trypan blue. Alive cells as indicated by no Trypan blue staining were counted under a microscope and our read-out normalised to this number. Data = luminescence RLU/number of live cells.

### Immunocytochemistry

Cells (2x10^4^ cells/well) were grown in Nunc Lab-Tek 8-chamber slides (Sigma, C7182, USA) and treated with the lipids as described in the figure legends. Cells were then fixed with 4% formaldehyde (Life Technologies, FB002, USA), permeabilised with 0.2% Triton X-100 (Sigma, T9284, USA), and incubated in primary antibodies diluted in blocking solution (2% bovine serum albumin (BSA) (Sigma, A6003, USA). Primary antibodies used were rabbit anti-cleaved caspase-3 (D175) (1:400, Cell Signalling Technology, 9661S, USA), rabbit anti-p62/SQSTM1 (1:500, Cell Signaling Technology, 4108, USA) subsequently incubated with secondary antibodies Alexa Fluor 594 goat anti-rabbit IgG (H+L) (1:500, Invitrogen, A-11037, USA). F-actin was visualised with Phalloidin-FITC (1:32, Sigma, P5282, USA) and nuclei with DAPI contained in Prolong mounting solution (Life Technologies, P36941, USA). Microscopy analyses were performed on a wide-field EVOS M5000 Cell Imaging microscope (Thermo Fisher, AMF5000, USA). Quantification of labelled cells was performed in at least 5 randomly selected high-power fields (60X magnification) per slide using the ImageJ Software (version v1.52a, National Institutes of Health, USA).

### Migration Assay

Myoblast migration assay was carried out as previously described (19, 20). Briefly, myoblasts (5 x 10^4^ cells/well) were seeded in 24-well-plates until reaching confluency and then incubated for 24 hours with DMEM containing 1% FBS. The cell monolayer was scratched with a pipette tip to obtain an acellular area in the middle of the well. Lipids were then added to the cells in a fresh medium with 1% FBS. Scratch width was measured using an EVOS M5000 Cell Imaging microscope (Thermo Fisher, AMF5000, USA). Myoblast migration at different incubation times was calculated as the percentage of scratch width over the initial scratch width.

### Myoblast Fusion Assay

Myoblast’s differentiation was induced for 5 days with or without lipids. Cells were then fixed in 100% cold methanol, and myotubes stained as previously described (21–23), with minor modifications. Briefly, cells were stained with May-Grunwald solution (Abcam, 150670, UK) for 15 minutes followed by Giemsa staining solution (Abcam, 150670, UK) for 30 minutes. Cells were visualized with the EVOS M5000 Cell Imaging microscope (Thermo Fisher, AMF5000, USA). Myotubes containing at least 3 nuclei were counted in 5 randomly selected regions per well using ImageJ software (version v1.52a, National Institutes of Health, USA).

### Glucose Uptake Assay

Glucose uptake was assessed in myotubes using the Glucose Uptake-Glo Assay (Promega, J1341, USA) following the manufacturer’s instructions. After three days of differentiation, myotubes were treated with either a control medium (no lipids) or medium with 0.5 μM/3 μM lipids, for two additional days. On the last day of treatment, serum was not added to the differentiation medium. Cells were then treated with or without 100 nM insulin for 2 hours at 37 °C, followed by the addition of 100 μM 2-deoxyglucose for 10 min at 37 °C. Glucose uptake was quantified recording luminescence with 0.3 to 1 second integration time using the Infinite 200 Pro microplate reader. Glucose uptake levels were then normalized to the number of live cells. After lipid treatment, wells were washed with 1X PBS, then 50 μL of Trypsin was added to the wells and incubated for 10 min. Trypsin reaction was stopped by adding 60 μL fresh 10% FBS DMEM (total volume = 110 μL). 10 μL of cell suspension was mixed with 10 μL of Trypan blue. Alive cells as indicated by no Trypan blue staining were counted under a microscope and our read-out normalised to this number. Data = luminescence RLU/number of live cells.

### Statistical Analysis

Each experiment was the average of three independent experiments. All treatments and time-points were performed in triplicates. All statistical analyses were completed using GraphPad Prism version 8.3.0 Statistics for Windows (GraphPad Software, San Diego, California USA). The effects of 1-DSA on cellular viability, metabolic activity, energy (ATP) production levels, migration, and the fusion of myoblasts were analysed by one-way ANOVA with 1-DSA treatment as factors. The effect of 1-DSA on glucose uptake of myotubes was analysed by two-way ANOVA with insulin effect and 1-DSA effect as factors. ANOVA results were then followed by a *post hoc* analysis using Tukey’s Honest Significant Difference test as appropriate. The effects of 1-DSA on inducing apoptosis, necrosis, and autophagy on myoblasts were analysed by two-tailed Student’s t-test. Data are presented as mean ± Standard Error of Mean (SEM). The results were considered statistically significant when *p* < 0.05.

## Results

### 1-DSA Treatment Reduces the Viability of Myoblasts

**Figure 1:**
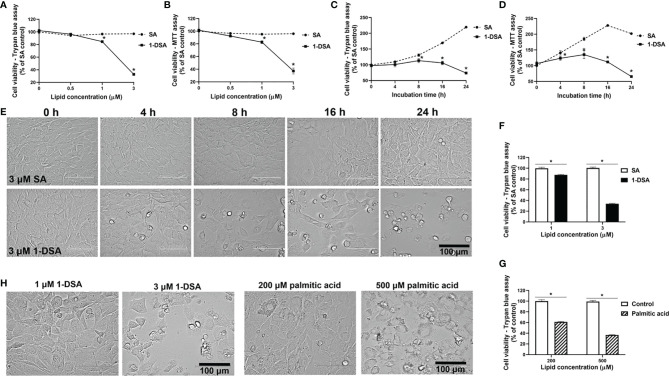
1-DSA reduce viable cell numbers in a concentration and time-dependent manner. **(A)** Cell viability of C2C12 myoblasts treated at 100% confluence with SA or 1-DSA at the selected concentrations and incubated for 24 hours and tested by trypan blue exclusion assay. SA groups were used as control. **(B)** Cell viability of C2C12 myoblasts treated with the designated SA or 1-DSA concentrations for 24 hours by MTT assay. **(C)** Cell viability of myoblasts treated with 3 μM SA or 3 μM 1-DSA at the indicated time points up to 24 hours by trypan blue exclusion assay. **(D)** Cell viability of C2C12 myoblasts treated with 3 μM SA or 3 μM 1-DSA at the indicated time points up to 24 hours by MTT assay. **(E)** Bright-field images showing C2C12 myoblast morphology treated at 100% confluence with 3 μM 1-DSA and 3 μM SA at indicated time points. **(F)** Cell viability of C2C12 myoblasts treated at 100% confluence with SA or 1-DSA at 1 µM and 3 µM and incubated for 24 hours, by trypan blue exclusion assay. Data are presented as mean ± SEM (n=3). *p < 0.05, SA *vs*. 1-DSA in **(A–D, F);** one-way ANOVA followed by *post hoc* tests. **(G)** Cell viability of C2C12 myoblasts treated at 100% confluence with control or palmitic acid at 200 µM and 500 µM and incubated for 24 hours, by trypan blue exclusion assay. Data are presented as mean ± SEM (n=3). *p < 0.05, control versus palmitic acid; one-way ANOVA followed by *post hoc* tests. **(H)** Representative cellular morphological images under the effect of 1-DSA (1 μM and 3 μM, respectively) and palmitic acid (200 μM and 500 μM, respectively) after 24 hour-incubation. Scale bars: 100 μm.

We investigated whether incubation with 1-DSL is directly toxic to C2C12 myoblasts, the most commonly used mouse skeletal muscle cell line (24, 25). Myoblasts were treated with low micromolar concentrations (0.5 µM, 1 µM, and 3 µM) of either the 1-DSL 1-deoxysphinganine (1-DSA) or the control sphinganine (SA). Incubation with 1-DSA significantly lowered the number of living cells in a concentration-dependent manner, as assessed by trypan blue exclusion assay (**Figure 1A**) and MTT assay (**Figure 1B**). There was no significant difference between the control SA groups at all the concentrations tested.

We then tested whether 1-DSA significantly reduced the number of live cells in a time-dependent manner. We measured cell viability over time upon incubation with 3 μM 1-DSA, a concentration that greatly reduced the number of live cells after 24 hours of treatment (See **Figures 1A, B**). Three micromoles of 1-DSA did not affect cell viability after 4 hrs of incubation, but significantly reduced the number of viable cells over the 24-hour-period, as determined by trypan blue exclusion assay (**Figure 1C**) and MTT assay (**Figure 1D**), which suggested that 1-DSL suppressed myoblast proliferation. A significant reduction in the number of live cells was accompanied by morphological alterations of C2C12 cells, including rounding up and detachment from the well surface (**Figure 1E**).

Lipid-induced toxicity has been demonstrated in muscle cells in the presence of saturated fatty acids (26). This lipotoxicity has the potential to induce insulin resistance in muscle cells (27, 28), and thus contribute to the pathophysiology of diabetes. We compared the lipotoxicity of the saturated fatty acid palmitic acid with 1-DSA. Treatment with 1-DSA (1 and 3 µM) significantly reduced the number of viable cells at concentrations significantly lower than palmitic acid (200 and 500 µM) (**Figures 1F, G**). Additionally, 1-DSA induced more cell rounding and detachment from the surface of the cell-culture flask, compared to the palmitic acid treatment (**Figure 1H**). This demonstrates that 1-DSA is much more cytotoxic to myoblasts than palmitic acid.

### 1-DSA Induce Necrosis, Apoptosis, and Autophagy and Raise ATP Levels in C2C12 Myoblasts

**Figure 2:**
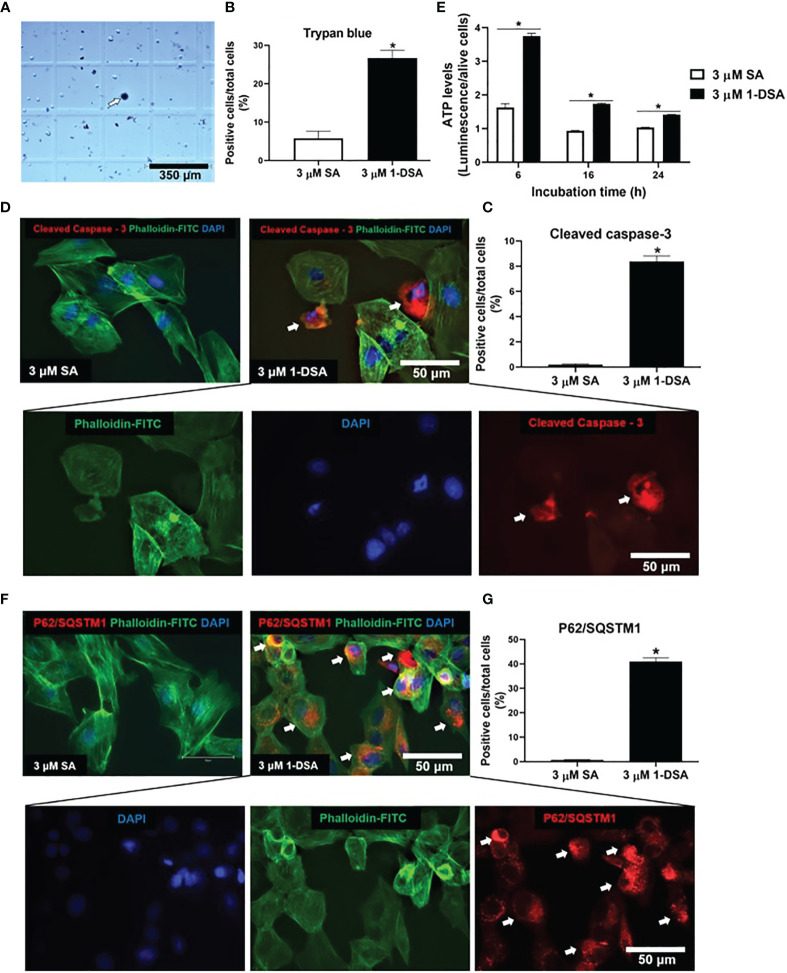
1-DSA induced necrosis, apoptosis, and autophagy in C2C12 cells. **(A)** Representative image of trypan blue stained myoblasts after 16-hour-treatment with either control 3 μM SA or 3 μM 1-DSA. The arrows depict positive necrotic stained cells. Scale bars, 350 μm. **(B)** Quantification of C2C12 necrotic dark blue dead cell numbers. **(C)** Quantification of positive stained apoptotic cells. **(D)** Representative images of C2C12 myoblast after 16-hour treatment with either control 3 μM SA or 3 μM 1-DSA, as shown by immunostaining with apoptotic marker Cleaved Caspase-3 (red), FITC-phalloidin (green), and DAPI (blue). Arrows indicate cleaved-caspase 3-positive cells. Scale bars, 50 μm. **(E)** Quantification of cellular ATP levels in C2C12 myoblasts with either 3 μM of SA or 1-DSA treatment at indicated time points. Data are presented as mean ± SEM (n=3). *p < 0.05, SA *vs*. 1-DSA; one-way ANOVA followed by *post hoc* tests. **(F)** 1-DSA induced autophagy as shown by immunostaining with autophagic marker P62/SQSTM1 (red), FITC-phalloidin (green), and DAPI (blue). Arrows indicate P62/SQSTM1-positive cells. Scale bars, 50 μm. **(G)** Quantification of positive stained autophagic cells. Data in **(B, D, G)** are presented as mean ± SEM (n=3); *p < 0.05, SA *vs*. 1-DSA; unpaired student’s t-test.

The reduction in live cell number observed upon 1-DSA treatment may be attributed to either reduced myoblast proliferation or increased cell death. The morphological changes described in (**Figures 1E, H**) suggest that incubation with the lipids induces cell death. This is in line with previous investigations in which an increase in ATP levels could be considered as pre-requisite of apoptotic processes (29, 30). This was further confirmed by quantification of dead cell numbers by trypan blue staining, showing that 3 μM of 1-DSA increased the number of trypan blue positive myoblasts (**Figures 2A, B**). Trypan blue staining relies on the loss of membrane integrity and is a strong indicator of cellular necrosis (31).

Immuno-staining for the apoptotic marker cleaved caspase-3 revealed that treatment with 3 μM of 1-DSA significantly induced apoptosis in C2C12 myoblasts (**Figure 2C**). Cleaved caspase-3 positive cells showed nuclear condensation and cell shrinkage, characteristic features of the apoptotic process (**Figure 2D**). To further investigate the induction of apoptosis upon 1-DSA incubation, we quantified cellular ATP content, as increased ATP is required to support the activity of hydrolytic enzymes, chromatin condensation, and formation of membrane-enclosed vesicles observed during apoptotic cell death (29). ATP levels were higher in 1-DSA treated cells at all the time points analysed (**Figure 2E**), further supporting that 1-DSA triggers apoptosis in C2C12 myoblasts.

Finally, we investigated the cellular processes underlying 1-DSA-mediated cytotoxicity. Autophagy is often associated with cell death (32, 33) and 1-DSA have recently been shown to increase the autophagic flux in embryonic fibroblasts and macrophages (34). Treatment with 3 μM of 1-DSA significantly increased the number of cells expressing the autophagic marker P62/SQSTM1 (**Figures 2F, G**), thus demonstrating that 1-DSA trigger autophagy in C2C12 myoblasts.

### 1-DSA Inhibit the Migration of C2C12 Myoblasts

**Figure 3:**
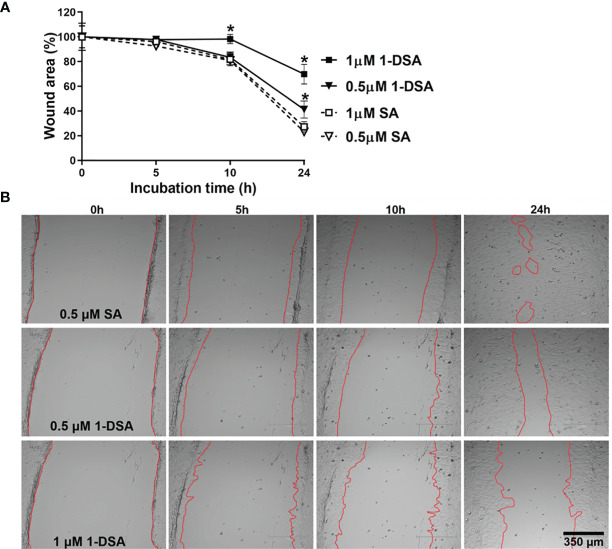
1-DSA significantly reduces the migration of muscle cells in a concentration and time-dependent manner, as tested by Wound-healing assay. **(A)** The wound-healing results are represented as the percentage of the initial wound area. Values are expressed as mean ± SEM (*n* = 3). *p < 0.05, SA *vs*. 1-DSA; one-way ANOVA followed by *post hoc* tests. **(B)** The representative wound-healing images of C2C12 myoblasts under the effect of either SA or 1-DSA were recorded by microscopy at the indicated time points. Scale bars: 350 μm.

Myoblast migration is critical for muscle integrity and regeneration as cell migration is the first step for myoblast differentiation (35, 36). Both myoblast migration and differentiation are impaired in T2DM (14, 17, 37–40), thus, we tested whether incubation with 1-DSA reduces the migration ability of C2C12 myoblasts. We incubated C2C12 myoblasts with 0.5 μM and 1 μM of 1-DSA, concentrations that induced only a minor decrease in the number of live cells and thus minimising the confounding element of cell death in our analysis. Our results showed that 1-DSA significantly reduced migration of myoblasts in a concentration-dependent and time-dependent manner, as measured by a wound-healing assay. (**Figures 3A, B**).

### 1-DSA Impair C2C12 Myoblasts Fusion Into Myotubes

**Figure 4:**
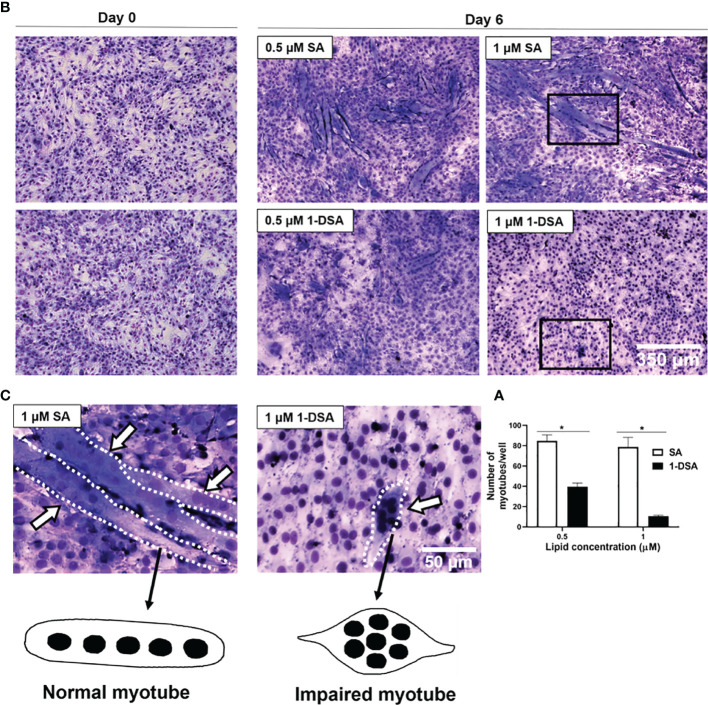
1-DSA inhibits myogenic differentiation and impaired myotube formation. **(A)** Quantification of myotubes per well after 5 days of SA or 1-DSA treatment. Values are expressed as mean ± SEM (*n* = 3). *p < 0.05, SA *vs*. 1-DSA; one-way ANOVA followed by *post hoc* tests. **(B)** Representative May-Grunwald Giemsa staining images of day 0 and day 6 of differentiation of C2C12 cells. Scale bars: 350 μm. **(C)** Insets represented a higher magnification of 5-day-differentiated myotubes treated with either 1 μM SA or 1-DSA. White arrows depict myotubes. Dotted lines cover the shape of myotubes. Scale bars: 50 μm.

After demonstrating that 1-DSA treatment reduces myoblast migration, we further evaluated whether it also reduces myoblast fusion into myotubes, a key step for the differentiation of myoblasts into mature myofibers. Treatment with 0.5 μM and 1 μM 1-DSA over 5 days reduced the number of myotubes in a concentration-dependent manner compared to the control group SA, as detected by May-Grunwald Giemsa staining (**Figures 4A, B**). Concomitant with lowering the frequency of cell fusion into myotubes, 1-DSA treatment also altered the morphology of myotubes. Specifically, in control SA treated cells, myotubes were elongated and contained nuclei distributed along the longitudinal axis. Conversely, in 1-DSA treated cells, the majority of the myotubes were shorter than controls and contained multiple nuclei concentrated in the centre of the expanded cell body (**Figure 4C**).

### 1-DSA Impair Insulin-Stimulated Glucose Uptake in C2C12 Myotubes

**Figure 5:**
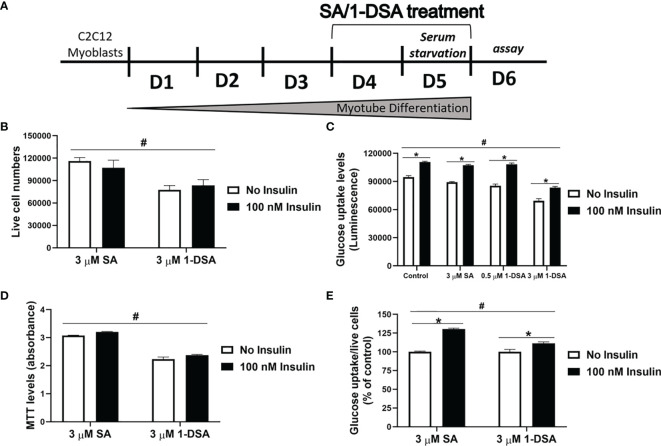
1-DSA induced insulin resistance by reducing glucose uptake in C2C12 5-day-differentiated-myotubes. **(A)** Model of C2C12 skeletal muscle differentiation and lipids SA or 1-DSA treatment for glucose uptake assay. SA was used as a control. **(B)** Cell viability of C2C12 myotubes treated with 3 μM SA or 1-DSA for 48 hours with/without insulin, as tested by trypan blue exclusion assay. No insulin groups were used as control. **(C)** Myotubes were treated with either 0 μM, 0.5 μM 1-DSA, or 3 μM SA/1-DSA with or without 100 nM insulin. Glucose uptake levels were determined with 2-DG by measuring luminescence, upon 48-hour- treatment with the lipids **(D)** Cell viability of C2C12 myotubes treated with 3 μM SA or 1-DSA for 48 hours with/without insulin, as tested by MTT assay. No insulin groups were used as control. **(E)** Quantification of glucose uptake level per live cell by normalizing luminescence to the number of live cells. Data are presented as a percentage of the control – SA/1-DSA no insulin group. Data correspond to the mean ± SEM (*n* = 3). *p < 0.05, no insulin *vs*. insulin; ^#^ *p* < 0.05, 1-DSA *vs*. SA; two-way ANOVA followed by *post hoc* tests.

Glucose intolerance is a characteristic feature of T2DM, where insulin-responsive tissues like skeletal muscle lose their ability to be stimulated by insulin and uptake glucose, leading to compromised glucose homeostasis (10, 41). To test whether 1-DSA impairs glucose uptake in muscle cells, we differentiated C2C12 myoblast into myotubes for 3 days and quantified glucose uptake after 2 days exposure with SA or 1-DSA (treatment regimen depicted in **Figure 5A**). Incubation with 1-DSA for this length of time did not alter cell viability (**Figure 5B**). We found that incubation with 3 μM of 1-DSA reduced the overall amount of glucose taken up by the cells, both in the absence and presence of insulin (**Figure 5C**). This effect was concentration-dependent, as 0.5 μM 1-DSA did not change the level of glucose uptake compared with control treatments. The reduction of glucose uptake in the presence of 1-DSA may derive from a lower number of live cells present upon lipid incubation. This was confirmed by a reduction in viable cell number in 1-DSA treated samples assessed by Trypan blue and MTT assays, respectively (**Figures 5B, D**). Finally, we investigated the insulin response at a cellular level by quantifying glucose uptake normalised by the number of viable cells. 1-DSA treatment reduced the magnitude of the insulin response in C2C12 myotubes (**Figure 5E**), suggesting that the lipid perturbs glucose uptake in myotubes.

## Discussion

Over the last 20 years, T2DM rates around the globe have risen dramatically and its consequences represent a major public health concern (42). In T2DM, the metabolism and functionality of skeletal muscle, an organ that plays a major role in maintaining blood glucose homeostasis, are compromised, and these deteriorations contribute to the progression of the disease (43, 44). Therefore, identifying the molecular factors that compromise skeletal muscle before and during T2DM is key to counteract the disease.

The findings of this study demonstrate that *in vitro* incubation with 1-DSA cause functional impairments in C2C12 skeletal muscle precursor and differentiated cells and that these impairments are similar to the ones observed *in vivo* and in T2DM patients, which have increased levels of circulating 1-DSL.

It has to be clarified that in patients treated with anti-diabetic medications (insulin and/or other ipoglicemic drugs) and/or with fasting plasma glucose levels ≥ 7mmol/L, the average of circulating 1-DSL plasma levels was 0.09 μM/L (3). As in T2DM several physiological responses can be highly responsive to environmental factors such as drugs and diet, and contingent on the cellular context, we decided to use concentrations of 1-DSL ranging from 0.5 μM, 1 μM, to 3 μM in line with the current literature (8). The present study confirmed the previously reported cytotoxic effect of 1-DSL (45–48) and importantly identifies mechanisms that may contribute to the development of T2DM.

### 1-DSA Compromises the Functionality of Precursor Myoblasts and Differentiated Myotubes

Muscle atrophy is typified by a reduction in myofiber size and muscle mass, and the resulting loss of muscle function as well as strength are commonly observed in T2DM patients (14, 49). In the current study, we found that low micromolar concentrations (1 µM and 3 μM) of 1-DSA cells reduced the number of live cells in both precursor and differentiated C2C12 cells. These findings support the notion that even relatively low concentrations of 1-DSL detected in the blood of MetS (pre-diabetes) patients (50) and T2DM patients (3) may be harmful to skeletal muscle cells. The plasma levels of 1-DSL were previously reported to significantly elevate in T2DM patients compared to the healthy group (3, 50). In addition, we showed that 1-DSA cytotoxicity is time-dependent, as the magnitude of the deleterious effects increased after prolonged lipid exposure. This is in accordance with the concept that, as a result of their biological structure, 1-DSL are metabolized intracellularly very slowly compared to typical sphingolipids, thus leading to a gradual accumulation of 1-DSL inside the cells and consequent cellular dysfunction (45).

Fast replicating, sub-confluent myoblasts were particularly sensitive to 1-DSA exposure, leading to cell death (**Supplementary Figure 1A**), while the effect was less pronounced in slowly replicating confluent cells and differentiated myotubes (**Supplementary Figure 1B**). Further investigations are required to determine the mechanisms underlying the different sensitivity observed. However, the lower replication rate of the cells following confluency and differentiation may account for the lower lipotoxicity, as previously shown in insulin-producing beta cells (8).

Additional analyses on the characteristics of 1-DSA-induced toxicity revealed that incubation with the lipid inhibited myoblast migration and myoblast differentiation into myotubes. These processes are crucial to maintain muscle homeostasis. Specifically, myoblast migration and differentiation are key steps in myogenesis and regeneration (51–53). During the regeneration phase, myoblasts migrate to the affected areas and undergo terminal differentiation where they align and fuse into myotubes (54, 55). The biochemical features of C2C12 myoblast cell line represents a meaningful and effective examination tool to examine skeletal muscle metabolism and differentiation (1, 2). We investigated common myogenic markers (MyoG, FABP3), and we found that 1-DSL did not alter their gene expression (**Supplementary Figure 2**). Further studies in myoblast primary cell lines and *in vivo* investigations would offer a better understanding of the gene and protein expression levels of myogenic markers for a targeted therapeutic intervention in patients with elevated 1-DSL levels. Our results also suggest that 1-DSA could contribute to the impaired myoblast differentiation and regeneration observed *in-vivo* during T2DM, which leads to skeletal muscle wasting and loss that often accompanies insulin resistance (56). In addition, our results indicate that 1-DSA incubation compromise glucose uptake in differentiated muscle cells. This has important implications in understanding the pathophysiology of T2DM, as it suggests that the effect of 1-DSA in skeletal muscle cells may contribute to the increase in blood levels of glucose observed in the disease.

We also reported that 1-DSA are much more cytotoxic to myoblasts than palmitic acid, a well-known saturated fatty acid causing insulin resistance in muscle cells (26, 27, 57). The saturated fatty acid palmitate also has a harmful effect on the myotube size and morphology of C2C12 myotubes (58). Palmitate-stimulated insulin resistance in C2C12 myotubes is closely associated with the reduction on myotube numbers and reduced expression of health benefit myokine genes, which confirmed the negative effect of palmitate accumulation in myotubes (59). Therefore, these findings highlight the concept that both 1-DSL and saturated fatty acids are responsible for muscle cell lipotoxicity, which is a key contributing factor in the pathophysiology of T2DM (60–62).

### Mechanisms of 1-DSA-Induced Lipotoxicity

Toxic effects induced by exposure to 1-DSA have been reported in different cell types, including insulin-producing β-cells (8), pancreatic acinar cells (63), and peripheral neurons (46, 48, 64), mouse embryonal fibroblasts (MEFs) (65), pig kidney epithelial cells and human prostate cancer cells (66). However, the underlying mechanisms of 1-DSA lipotoxicity have not been fully elucidated. Some studies reported that 1-DSA incubation damages the structure and functionality of intracellular organelles, including mitochondria (67), ER (65), and Golgi apparatus (34). Damaged or stressed organelles are normally degraded and recycled *via* the cellular process of autophagy (68). In addition to the previous study of Lauterbach et al., 1-DSA were recently found to alter cellular autophagy and to induce autophagosome accumulation in MEFs (34). Our data showed that exposure to 1-DSA increase autophagosomes formation in C2C12 myoblasts and altered autophagy activity is one of the major causes for a variety of skeleton muscle disorders (69). This is of special interest as dysfunction in the autophagic process is linked with the development of obesity and T2DM (13, 69). As plasma levels of 1-DSL are elevated in metabolic MetS and T2DM patients (2, 3), our findings suggest a potential involvement of 1-DSL in activating autophagosome in the context of the disease. Further investigations are required to elucidate the molecular mechanisms by which exposure to 1-DSA trigger autophagy. One possible cellular mediator for the observed phenotype is the generation of 1-deoxyceramide, a downstream metabolite of 1-DSA that accumulates in cells upon 1-DSA treatment *in vitro* (70). In addition, 1-deoxyceramide is highly enriched *in vivo* in visceral adipose tissue as well as in the serum of obese patients with T2DM (71). Ceramides are bioactive signalling molecules that counteract the activity of suppressors of autophagy, including Class I PI3K and AKT, *via* activation of PP2A (72). Ceramides have also been proven to alleviate IL-13-mediated inhibition of autophagy (73), which is believed to require the class I PI3K/AKT pathway (74). 1-DSA-derived 1-deoxyceramide may also contribute to the apoptotic death observed in skeletal muscle cells. In support of this hypothesis, ceramides can trigger caspases 3 and 7 and thus stimulate apoptosis (75). Ceramides can also alter the permeability of the outer membrane of mitochondria (75), by forming channels through the mitochondrial membrane (76), which is an critical step in the generation of apoptosis.

## Conclusion

The findings of this study revealed that 1-DSA exerts cytotoxic effects in both myoblast progenitors and differentiated myotubes. Our study suggests that the risen levels of 1-DSL detected in T2DM patients may contribute to the diminished functionality of skeletal muscle tissue. Further studies are required to further validate our findings in primary muscle cells, human skeletal muscle organoids and mouse models. In addition, as circulating levels of 1-DSL can be modulated *via* dietary interventions (47, 63), additional investigations are warranted to test whether lowering 1-DSL may improve glycaemic control and then complement presently available therapies in preventing T2DM.

## Data Availability Statement

The raw data supporting the conclusions of this article will be made available by the authors, without undue reservation.

## Author Contributions

This study was designed and coordinated by SS. Laboratory investigations and statistical analyses were performed by DT. The manuscript was designed and prepared by DT, VC, and SS. Manuscript was revised and commented by SM, RE, CM, and DH. All authors contributed to the article and approved the submitted version.

## Conflict of Interest

The authors declare that the research was conducted in the absence of any commercial or financial relationships that could be construed as a potential conflict of interest.

## Publisher’s Note

All claims expressed in this article are solely those of the authors and do not necessarily represent those of their affiliated organizations, or those of the publisher, the editors and the reviewers. Any product that may be evaluated in this article, or claim that may be made by its manufacturer, is not guaranteed or endorsed by the publisher.

## Supplementary Material

Supplementary Figure 1 1-DSA reduce viable cell numbers in a concentration-dependent manner. **(A)** C2C12 cells were seeded at 5 x 10^3^ cells/well. Cell viability of C2C12 myoblasts treated at sub-confluence with SA or 1-DSA at the selected concentrations and incubated for 24 h, as tested by MTT assay. SA groups were used as control. **(B)** C2C12 cells were seeded at 2 x 104 cells/well. Cell viability of C2C12 5-day-differentiated myotubes, included 24 h of SA or 1-DSA treatment at the selected concentrations, as tested by MTT assay. Data were presented as mean ± SEM (n=3). *p < 0.05, SA *vs*. 1-DSA; one-way ANOVA followed by *post hoc* tests.

Supplementary Figure 2 Effect of 1-DSA on mRNA expression of myogenic markers. mRNA levels were normalized to EEF2 mRNA and presented as fold change.

## Abbreviations

1-DSA, 1-deoxysphinganine; 1-DSL, 1-deoxysphingolipids; ATP, Adenosine Triphosphate; MTT, Thiazolyl Blue Tetrazolium Bromide; PA, palmitic acid; SA, sphinganine; SL, sphingolipids; T2DM, Type 2 Diabetes Mellitus.

## References

[1] Bertea M Rütti MF Othman A Marti-Jaun J Hersberger M von Eckardstein A. Deoxysphingoid bases as plasma markers in diabetes mellitus. Lipids Health Dis (2010) 9:84. doi: 10.1186/1476-511x-9-84 20712864 PMC2931514

[2] Othman A Rutti MF Ernst D Saely CH Rein P Drexel H. Plasma deoxysphingolipids: a novel class of biomarkers for the metabolic syndrome? Diabetologia (2012) 55(2):421–31. doi: 10.1007/s00125-011-2384-1 22124606

[3] Mwinyi J Boström A Fehrer I Othman A Waeber G Marti-Soler H. Plasma 1-deoxysphingolipids are early predictors of incident type 2 diabetes mellitus. PloS One (2017) 12(5):175776. doi: 10.1371/journal.pone.0175776 PMC5417440 28472035

[4] Wei N Pan J Pop-Busui R Othman A Alecu I Hornemann T. Altered sphingoid base profiles in type 1 compared to type 2 diabetes. Lipids Health Dis (2014) 13(1):161. doi: 10.1186/1476-511X-13-161 25305670 PMC4271467

[5] Zarini S Perreault L Newsom SA Kahn DE Kerege A Harrison KA. eoxysphingolipids—Novel Skeletal Muscle Lipids Related to Insulin Resistance in Humans That Decrease Insulin Sensitivity In Vitro. Am Diabetes Assoc (2018). doi: 10.2337/db18-1935-P

[6] Gault CR Obeid LM Hannun YA. An overview of sphingolipid metabolism: from synthesis to breakdown. Adv Exp Med Biol (2010) 688:1–23. doi: 10.1007/978-1-4419-6741-442 1_1 20919643 10.1007/978-1-4419-6741-1_1 PMC3069696

[7] Hannun YA Obeid LM. Principles of bioactive lipid signalling: lessons from sphingolipids. Nat Rev Mol Cell Biol (2008) 9:139. doi: 10.1038/nrm2329 18216770

[8] Zuellig RA Hornemann T Othman A Hehl AB Bode H Güntert T. Deoxysphingolipids, novel biomarkers for type 2 diabetes, are cytotoxic for insulin-producing cells. Diabetes (2014) 63(4):1326–39. doi: 10.2337/db13-1042 24379346

[9] Dohrn MF Othman A Hirshman SK Bode H Alecu I Fähndrich E. Elevation of plasma 1-deoxy-sphingolipids in type 2 diabetes mellitus: a susceptibility to neuropathy? Eur J Neurol (2015) 22(5):806–14, e55. doi: 10.1111/ene.12663 25623782

[10] DeFronzo RA Tripathy D. Skeletal muscle insulin resistance is the primary defect in type 2 diabetes. Diabetes Care (2009) 32(suppl 2):157–63. doi: 10.2337/dc09-S302 PMC2811436 19875544

[11] Park SW Goodpaster BH Strotmeyer ES de Rekeneire N Harris TB Schwartz AV. Decreased muscle strength and quality in older adults with type 2 diabetes: the health, aging, and body composition study. Diabetes (2006) 55(6):1813–8. doi: 10.2337/db05-1183 16731847

[12] Sayer AA Dennison EM Syddall HE Gilbody HJ Phillips DI Cooper C. Type 2 diabetes, muscle strength, and impaired physical function: the tip of the iceberg? Diabetes Care (2005) 28(10):2541–2. doi: 10.2337/diacare.28.10.2541 16186295

[13] Henriksen T Wigge L Nielsen J Pedersen B Sandri M Scheele C. Dysregulated autophagy in muscle precursor cells from humans with type 2 diabetes. Sci Rep (2019) 9(1):1–11. doi: 10.1038/s41598-019-44535-2 31160616 PMC6546785

[14] Teng S Huang P. The effect of type 2 diabetes mellitus and obesity on muscle progenitor cell function. Stem Cell Res Ther (2019) 10(1):103. doi: 10.1186/s13287-019-1186-0 30898146 PMC6427880

[15] D'Souza DM Al-Sajee D Hawke TJ. Diabetic myopathy: impact of diabetes mellitus on skeletal muscle progenitor cells. Front Physiol (2013) 4:379. doi: 10.3389/fphys.2013.00379 24391596 PMC3868943

[16] Vignaud A Ramond F Hourde C Keller A Butler-Browne G Ferry A. Diabetes provides an unfavorable environment for muscle mass and function after muscle injury in mice. Pathobiology (2007) 74(5):291–300. doi: 10.1159/000105812 17890896

[17] Fiaschi T Tedesco FS Giannoni E Diaz-Manera J Parri M Cossu G. Globular adiponectin as a complete mesoangioblast regulator: role in proliferation, survival, motility, and skeletal muscle differentiation. Mol Biol Cell (2010) 21(6):848–59. doi: 10.1091/mbc.e09-04-0310 PMC2836966 20089845

[18] Hommelberg PP Plat J Langen R Schols A Mensink R. Fatty acid-induced NF-κB activation and insulin resistance in skeletal muscle are chain length dependent. Am J Physiology-Endocrinology Metab (2009) 296(1):E114–E20. doi: 10.1152/ajpendo.00436.2007 18957619

[19] Yeow K Cabane C Turchi L Ponzio G Derijard B. Increased MAPK signaling during *in vitro* muscle wounding. Biochem Biophys Res Commun (2002) 293(1):112–9. doi: 10.1016/S0006-291X(02)00190-0 12054571

[20] Ko MH Li CY Lee CF Chang CK Fang SH. Scratch wound closure of myoblasts and myotubes is reduced by inflammatory mediators. Int Wound J (2016) 13(5):680–5. doi: 10.1111/iwj.12346 PMC7949697 25123045

[21] Tanaka S Ono Y Sakamoto K. DCEBIO facilitates myogenic differentiation *via* intermediate conductance Ca2+ activated K+ channel activation in C2C12 myoblasts. J Pharmacol Sci (2017) 133(4):276–9. doi: 10.1016/j.jphs.2017.02.005 28302447

[22] Fortier M Comunale F Kucharczak J Blangy A Charrasse S Gauthier-Rouvière C. RhoE controls myoblast alignment prior fusion through RhoA and ROCK. Cell Death Differ (2008) 15(8):1221–31. doi: 10.1038/cdd.2008.34 18369372

[23] Sestili P Barbieri E Martinelli C Battistelli M Guescini M Vallorani L. Creatine supplementation prevents the inhibition of myogenic differentiation in oxidatively injured C2C12 murine myoblasts. Mol Nutr Food Res (2009) 53(9):1187–204. doi: 10.1002/mnfr.200800504 19653222

[24] Rodgers BD Wiedeback BD Hoversten KE Jackson MF Walker RG Thompson TB. Myostatin stimulates, not inihibits, C2C12 myoblast proliferation. Endocrinology (2014) 155(3):670–5. doi: 10.1210/en.2013-2107 PMC3929746 24424069

[25] Uemura K Hayashi M Itsubo T Oishi A Iwakawa H Komatsu M. Myostatin promotes tenogenic differentiation of C2C12 myoblast cells through Smad3. FEBS Open Bio (2017) 7(4):522–32. doi: 10.1002/2211-5463.12200 PMC5377394 28396837

[26] Hirabara SM Curi R Maechler P. Saturated fatty acid-induced insulin resistance is associated with mitochondrial dysfunction in skeletal muscle cells. J Cell Physiol (2010) 222(1):187–94. doi: 10.1002/jcp.21936 19780047

[27] Chen X Xu S Wei S Deng Y Li Y Yang F. Comparative Proteomic Study of Fatty Acid-treated Myoblasts Reveals Role of Cox-2 in Palmitate-induced Insulin Resistance. Sci Rep (2016) 6(1):21454. doi: 10.1038/srep21454 26899878 PMC4761885

[28] Tong T Ren N Soomi P Wu J Guo N Kang H. Theaflavins improve insulin sensitivity through regulating mitochondrial biosynthesis in palmitic acid-induced HepG2 cells. Molecules (2018) 23(12):3382. doi: 10.3390/molecules23123382 PMC6320999 30572687

[29] Kakarla R Hur J Kim J Kim J Chwae J. Apoptotic cell-derived exosomes: Messages from dying cells. Exp Mol Med (2020), 1–6. doi: 10.1038/s12276-019-0362-8 31915368 PMC7000698

[30] Atlante A Giannattasio S Bobba A Gagliardi S Petragallo V Calissano P. An increase in the ATP levels occurs in cerebellar granule cells en route to apoptosis in which ATP derives from both oxidative phosphorylation and anaerobic glycolysis. Biochim Biophys Acta (2005) 1708(1):50–62. doi: 10.1016/j.bbabio.2005.01.009 15949983

[31] Zhivotosky B Orrenius S. Assessment of apoptosis and necrosis by DNA fragmentation and morphological criteria. Curr Protoc Cell Biol (2001) 12(1):18. doi: 10.1002/0471143030.cb1803s12 18228342

[32] Lane JD Yonekawa T Thorburn A. Autophagy and cell death. Essays Biochem (2013) 55:105–17. doi: 10.1042/bse0550105 PMC3894632 24070475

[33] Minina EA Bozhkov PV Hofius D. Autophagy as initiator or executioner of cell death. Trends Plant Sci (2014) 19(11):692–7. doi: 10.1016/j.tplants.2014.07.007 25156061

[34] Lauterbach MA Saavedra V Mangan MS Penno A Thiele C Latz E. 1-Deoxysphingolipids cause autophagosome and lysosome accumulation and trigger NLRP3 inflammasome activation. Autophagy (2020), 1–15. doi: 10.1080/15548627.2020.1804677 PMC8386713 32835606

[35] McNally EM Pytel P. Muscle diseases: the muscular dystrophies. Annu Rev Pathol Mech Dis (2007) 2:87–109. doi: 10.1146/annurev.pathol.2.010506.091936 18039094

[36] Tannu NS Rao VK Chaudhary RM Giorgianni F Saeed AE Gao Y. Comparative proteomes of the proliferating C2C12 myoblasts and fully differentiated myotubes reveal the complexity of the skeletal muscle differentiation program. Mol Cell Proteomics (2004) 3(11):1065–82. doi: 10.1074/mcp.M400020-MCP200 15286212

[37] Aragno M Mastrocola R Catalano MG Brignardello E Danni O Boccuzzi G. Oxidative stress impairs skeletal muscle repair in diabetic rats. Diabetes (2004) 53(4):1082–8. doi: 10.2337/diabetes.53.4.1082 15047625

[38] Nguyen M-H Cheng M Koh TJ. Impaired muscle regeneration in ob/ob and db/db mice. Sci World J (2011) 11:1525–35. doi: 10.1100/tsw.2011.137 PMC5720064 21805021

[39] Lerman OZ Galiano RD Armour M Levine JP Gurtner GC. Cellular dysfunction in the diabetic fibroblast: impairment in migration, vascular endothelial growth factor production, and response to hypoxia. Am J Pathol (2003) 162(1):303–12. doi: 10.1016/S0002-9440(10)63821-7 PMC1851127 12507913

[40] Capla JM Grogan RH Callaghan MJ Galiano RD Tepper OM Ceradini DJ. Diabetes impairs endothelial progenitor cell–mediated blood vessel formation in response to hypoxia. Plast Reconstr Surg (2007) 119(1):59–70. doi: 10.1097/01.prs.0000244830.16906.3f 17255657

[41] Khan A Pessin J. Insulin regulation of glucose uptake: a complex interplay of intracellular signalling pathways. Diabetologia (2002) 45(11):1475–83. doi: 10.1007/s00125-002-0974-7 12436329

[42] Ginter E Simko V. Type 2 diabetes mellitus, pandemic in 21st century. Diabetes (2013), 42–50. doi: 10.1007/978-1-4614-5441-0_6 23393670

[43] Petersen KF Shulman GI. Pathogenesis of skeletal muscle insulin resistance in type 2 diabetes mellitus. Am J Cardiol (2002) 90(5):11–8. doi: 10.1016/S0002-9149(02)02554-7 12231074

[44] Bouzakri K Koistinen HA Zierath JR. Molecular mechanisms of skeletal muscle insulin resistance in type 2 diabetes. Curr Diabetes Rev (2005) 1(2):167–74. doi: 10.2174/1573399054022785 18220592

[45] Alecu I Othman A Penno A Saied EM Arenz C von Eckardstein A. Cytotoxic 1-deoxysphingolipids are metabolized by a cytochrome P450-dependent pathway. J Lipid Res (2017) 58(1):60–71. doi: 10.1194/jlr.M072421 27872144 PMC5234722

[46] Kramer R Bielawski J Kistner-Griffin E Othman A Alecu I Ernst D. Neurotoxic 1-deoxysphingolipids and paclitaxel-induced peripheral neuropathy. FASEB J (2015) 29(11):4461–72. doi: 10.1096/fj.15-272567 PMC4608911 26198449

[47] Othman A Bianchi R Alecu I Wei Y Porretta-Serapiglia C Lombardi R. Lowering Plasma 1-Deoxysphingolipids Improves Neuropathy in Diabetic Rats. Diabetes (2015) 64(3):1035–45. doi: 10.2337/db14-1325 25277395

[48] Penno A Reilly MM Houlden H Laurá M Rentsch K Niederkofler V. Hereditary sensory neuropathy type 1 is caused by the accumulation of two neurotoxic sphingolipids. J Biol Chem (2010) 285(15):11178–87. doi: 10.1074/jbc.M109.092973 PMC2856995 20097765

[49] Park SW Goodpaster BH Lee JS Kuller LH Boudreau R De Rekeneire N. Excessive loss of skeletal muscle mass in older adults with type 2 diabetes. Diabetes Care (2009) 32(11):1993–7. doi: 10.2337/dc09-0264 PMC2768193 19549734

[50] Othman A Saely CH Muendlein A Vonbank A Drexel H von Eckardstein A. Plasma 1-deoxysphingolipids are predictive biomarkers for type 2 diabetes mellitus. BMJ Open Diabetes Res Care (2015) 3(1):73. doi: 10.1136/bmjdrc-2014-000073 PMC4368929 25815206

[51] Ferrari G Angelis D Coletta M Paolucci E Stornaiuolo A Cossu G. Muscle regeneration by bone marrow-derived myogenic progenitors. Science (1998) 279(5356):1528–30. doi: 10.1126/science.279.5356.1528 9488650

[52] Yusuf F Brand-Saberi B. Myogenesis and muscle regeneration. Histochem Cell Biol (2012) 138(2):187–99. doi: 10.1007/s00418-012-0972-x 22644378

[53] Nishimura T Nakamura K Kishioka Y Kato-Mori Y Wakamatsu J-i Hattori A. Inhibition of matrix metalloproteinases suppresses the migration of skeletal muscle cells. J Muscle Res Cell Motil (2008) 29(1):37–44. doi: 10.1007/s10974-008-9140-2 18563597

[54] Morgan JE Partridge TA. Muscle satellite cells. Int J Biochem Cell Biol (2003) 35(8):1151–6. doi: 10.1016/S1357-2725(03)00042-6 12757751

[55] Le Grand F Rudnicki MA. Skeletal muscle satellite cells and adult myogenesis. Curr Opin Cell Biol (2007) 19(6):628–33. doi: 10.1016/j.ceb.2007.09.012 PMC2215059 17996437

[56] Wang X Hu Z Hu J Du J Mitch WE. Insulin resistance accelerates muscle protein degradation: activation of the ubiquitin-proteasome pathway by defects in muscle cell signaling. Endocrinology (2006) 147(9):4160–8. doi: 10.1210/en.2006-0251 16777975

[57] Wang X Yu W Nawaz A Guan F Sun S Wang C. Palmitate Induced Insulin Resistance by PKCtheta-Dependent Activation of mTOR/S6K Pathway in C2C12 Myotubes. Exp Clin Endocrinol Diabetes (2010) 118(09):657–61. doi: 10.1055/s-0030-1252069 20429048

[58] Bryner RW Woodworth-Hobbs ME Williamson DL Alway SE. Docosahexaenoic acid protects muscle cells from palmitate-induced atrophy. Int Scholarly Res Notices (2012) 2012:647348–62. doi: 10.5402/2012/647348 PMC3914282 24533207

[59] Yang M Wei D Mo C Zhang J Wang X Han X. Saturated fatty acid palmitate-induced insulin resistance is accompanied with myotube loss and the impaired expression of health benefit myokine genes in C2C12 myotubes. Lipids Health Dis (2013) 12(1):1–10. doi: 10.1186/1476-511X-12-104 23866690 PMC3723881

[60] Meex RC Blaak EE van Loon LJ. Lipotoxicity plays a key role in the development of both insulin resistance and muscle atrophy in patients with type 2 diabetes. Obes Rev (2019) 20(9):1205–17. doi: 10.1111/obr.12862 PMC6852205 31240819

[61] Oh YS Bae GD Baek DJ Park E-Y Jun H-S. Fatty acid-induced lipotoxicity in pancreatic beta-cells during development of type 2 diabetes. Front Endocrinol (Lausanne) (2018) 9:384. doi: 10.3389/fendo.2018.00384 30061862 PMC6054968

[62] Brøns C Grunnet LG. Mechanisms in endocrinology: skeletal muscle lipotoxicity in insulin resistance and type 2 diabetes: a causal mechanism or an innocent bystander? Eur J Endocrinol (2017) 176(2):67–78. doi: 10.1530/eje-16-0488 27913612

[63] Chen R Hornemann T Štefanić S Schraner EM Zuellig R Reding T. Serine administration as a novel prophylactic approach to reduce the severity of acute pancreatitis during diabetes in mice. Diabetologia (2020) 63:1885–99. doi: 10.1007/s00125-020-05156-x 32385601

[64] Becker KA Uerschels AK Goins L Doolen S McQuerry KJ Bielawski J. Role of 1-Deoxysphingolipids in docetaxel neurotoxicity. J Neurochem (2020) 154(6):662–72. doi: 10.1111/jnc.14985 PMC7426245 32058598

[65] Alecu I Tedeschi A Behler N Wunderling K Lamberz C Lauterbach MR. Localization of 1-deoxysphingolipids to mitochondria induces mitochondrial dysfunction. J Lipid Res (2017) 58(1):42–59. doi: 10.1194/jlr.M068676 27881717 PMC5234710

[66] Zitomer NC Mitchell T Voss KA Bondy GS Pruett ST Garnier-Amblard EC. Ceramide synthase inhibition by fumonisin B1 causes accumulation of 1-deoxysphinganine: a novel category of bioactive 1-deoxysphingoid bases and 1-deoxydihydroceramides biosynthesized by mammalian cell lines and animals. J Biol Chem (2009) 284(8):4786–95. doi: 10.1074/jbc.M808798200 PMC2643501 19095642

[67] Wilson ER Kugathasan U Abramov AY Clark AJ Bennett DL Reilly MM. Hereditary sensory neuropathy type 1-associated deoxysphingolipids cause neurotoxicity, acute calcium handling abnormalities and mitochondrial dysfunction in vitro. Neurobiol Dis (2018) 117:1–14. doi: 10.1016/j.nbd.2018.05.008 29778900 PMC6060082

[68] Klionsky DJ Abdelmohsen K Abe A Abedin MJ Abeliovich H Acevedo Arozena A. Guidelines for the use and interpretation of assays for monitoring autophagy. autophagy (2016) 12(1):1–222. doi: 10.1080/15548627.2020.1797280 26799652 PMC4835977

[69] Xia Q Huang X Huang J Zheng Y March ME Li J. The Role of Autophagy in Skeletal Muscle Diseases. Front Physiol (2021) 12:638983(291). doi: 10.3389/fphys.2021.638983 33841177 PMC8027491

[70] Schwartz NU Mileva I Gurevich M Snider J Hannun YA Obeid LM. Quantifying 1-deoxydihydroceramides and 1-deoxyceramides in mouse nervous system tissue. Prostaglandins Other Lipid Mediat (2019) 141:40–8. doi: 10.1016/j.prostaglandins.2019.02.005 PMC6467697 30790665

[71] Hannich JT Loizides-Mangold U Sinturel F Harayama T Vandereycken B Saini C. Ether lipids, sphingolipids, and toxic 1-deoxyceramides as hallmarks for lean and obese type 2 diabetic patients. Acta Physiologica (2020), 13610. doi: 10.1111/apha.13610 33351229

[72] Scarlatti F Bauvy C Ventruti A Sala G Cluzeaud F Vandewalle A. Ceramide-mediated macroautophagy involves inhibition of protein kinase B and up-regulation of beclin 1. J Biol Chem (2004) 279(18):18384–91. doi: 10.1074/jbc.M313561200 14970205

[73] Petiot A Ogier-Denis E Blommaart EF Meijer AJ Codogno P. Distinct classes of phosphatidylinositol 3′-kinases are involved in signaling pathways that control macroautophagy in HT-29 cells. J Biol Chem (2000) 275(2):992–8. doi: 10.1074/jbc.275.2.992 10625637

[74] Lavieu G Scarlatti F Sala G Carpentier S Levade T Ghidoni R. Regulation of autophagy by sphingosine kinase 1 and its role in cell survival during nutrient starvation. J Biol Chem (2006) 281(13):8518–27. doi: 10.1074/jbc.M506182200 16415355

[75] Mullen TD Hannun YA Obeid LM. Ceramide synthases at the centre of sphingolipid metabolism and biology. Biochem J (2012) 441(3):789–802. doi: 10.1042/BJ20111626 22248339 PMC3689921

[76] Colombini M. Ceramide channels and their role in mitochondria-mediated apoptosis. Biochim Biophys Acta (BBA)-Bioenergetics (2010) 1797(6-7):1239–44. doi: 10.1016/j.bbabio.2010.01.021 20100454

