# Supplementary figures and images for: 1-Deoxysphingolipids, Early Predictors of Type 2 Diabetes, Compromise the Functionality of Skeletal Myoblasts
Source: Front Endocrinol (Lausanne). 2021 Dec 24;12:772925. doi: 10.3389/fendo.2021.772925 (PMC8739520; doi:10.3389/fendo.2021.772925)

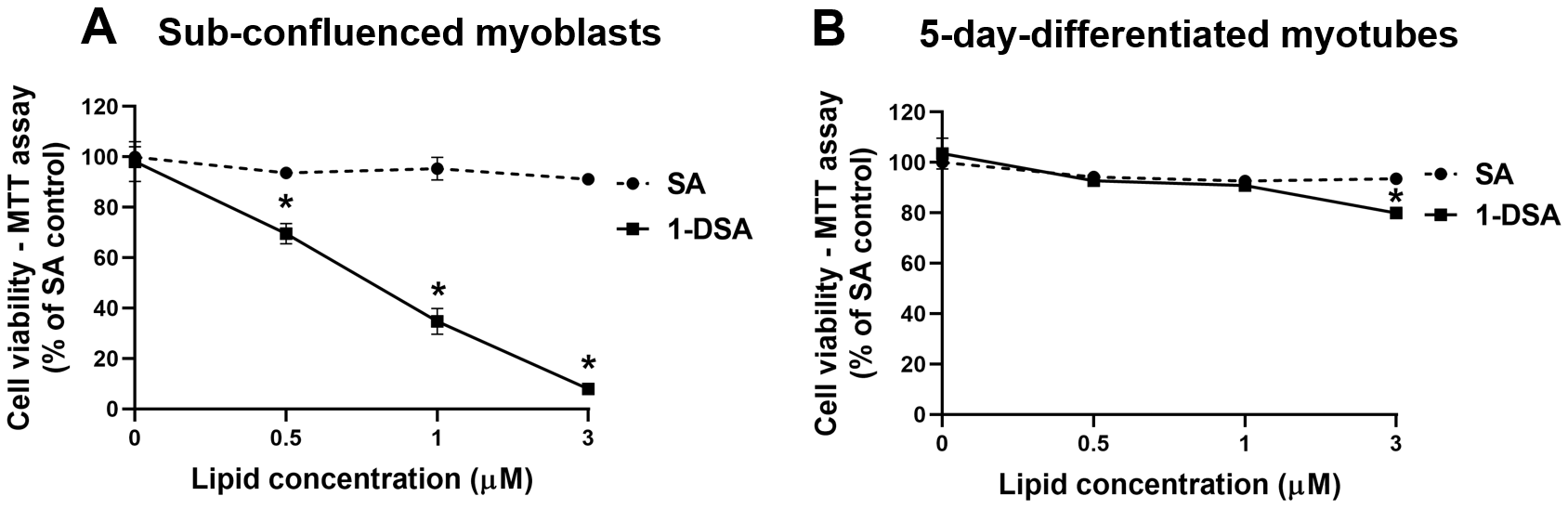

Supplement: Supplementary Figure 1 — 1-DSA reduce viable cell numbers in a concentration-dependent manner. (A) C2C12 cells were seeded at 5 x 10^3^ cells/well. Cell viability of C2C12 myoblasts treated at sub-confluence with SA or 1-DSA at the selected concentrations and incubated for 24 h, as tested by MTT assay. SA groups were used as control. (B) C2C12 cells were seeded at 2 x 10^4^ cells/well. Cell viability of C2C12 5-day-differentiated myotubes, included 24 h of SA or 1-DSA treatment at the selected concentrations, as tested by MTT assay. Data were presented as mean ± SEM (n=3). *p < 0.05, SA vs. 1-DSA; one-way ANOVA followed by post hoc tests. [file Image_1.tif]

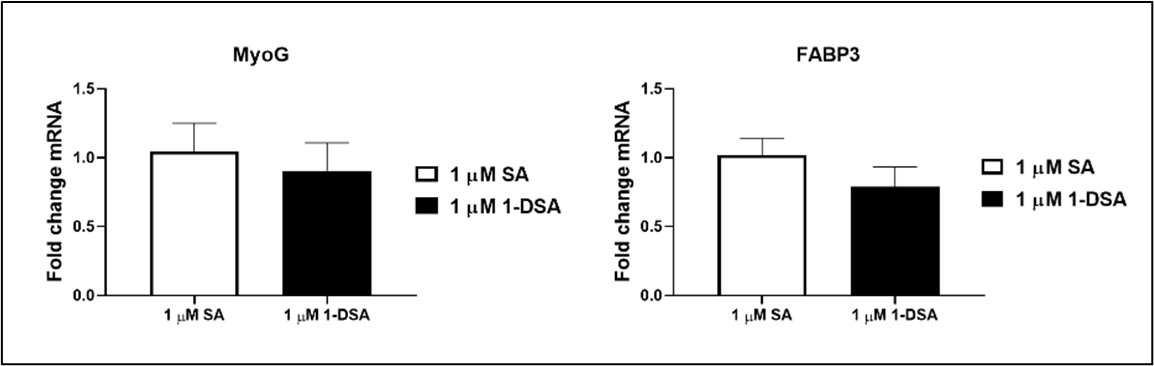

Supplement: Supplementary Figure 2 — Effect of 1-DSA on mRNA expression of myogenic markers. mRNA levels were normalized to EEF2 mRNA and presented as fold change. [file Image_2.tif]
